# Measurement and Study of Lidar Ratio by Using a Raman Lidar in Central China

**DOI:** 10.3390/ijerph13050508

**Published:** 2016-05-18

**Authors:** Wei Wang, Wei Gong, Feiyue Mao, Zengxin Pan, Boming Liu

**Affiliations:** 1 State Key Laboratory of Information Engineering in Surveying, Mapping and Remote Sensing (LIESMARS), Wuhan University, Wuhan 430079, China; wangweicn@whu.edu.cn (W.W.); pzx@whu.edu.cn (Z.P.); liuboming@whu.edu.cn (B.L.); 2 Collaborative Innovation Center for Geospatial Technology, Wuhan 430079, China; 3 Hubei Collaborative Innovation Center for High-Efficiency Utilization of Solar Energy, Wuhan 430068, China; 4 School of Remote Sensing and Information Engineering, Wuhan University, Wuhan 430079, China

**Keywords:** lidar ratio, aerosol, wind, monsoon

## Abstract

We comprehensively evaluated particle lidar ratios (*i.e.*, particle extinction to backscatter ratio) at 532 nm over Wuhan in Central China by using a Raman lidar from July 2013 to May 2015. We utilized the Raman lidar data to obtain homogeneous aerosol lidar ratios near the surface through the Raman method during no-rain nights. The lidar ratios were approximately 57 ± 7 sr, 50 ± 5 sr, and 22 ± 4 sr under the three cases with obviously different pollution levels. The haze layer below 1.8 km has a large particle extinction coefficient (from 5.4e-4 m^−1^ to 1.6e-4 m^−1^) and particle backscatter coefficient (between 1.1e-05 m^−1^sr^−1^ and 1.7e-06 m^−1^sr^−1^) in the heavily polluted case. Furthermore, the particle lidar ratios varied according to season, especially between winter (57 ± 13 sr) and summer (33 ± 10 sr). The seasonal variation in lidar ratios at Wuhan suggests that the East Asian monsoon significantly affects the primary aerosol types and aerosol optical properties in this region. The relationships between particle lidar ratios and wind indicate that large lidar ratio values correspond well with weak winds and strong northerly winds, whereas significantly low lidar ratio values are associated with prevailing southwesterly and southerly wind.

## 1. Introduction

Atmospheric aerosols significantly influence the radiation budget of the Earth according to the effect of precipitation rates, the lifetime and microphysical properties of clouds, and tropospheric photochemistry; therefore, they are significant in climate change studies [1,2]. However, numerous issues continue to restrict the understanding of their chemical and physical properties and their spatiotemporal distribution characteristics [3]. Our limited understanding of aerosol vertical characteristics is ascribed to their high variation in space-time [4]. With the rapid growth of the Chinese economy in recent years, air pollution has reached a critical level [5,6,7]. The aerosol lidar is the most widely utilized tool to measure aerosol vertical properties, but the retrieval process requires an assumptive lidar ratio (*i.e.*, particle extinction to backscatter ratio), which may result in a large error. Lidar ratio also contains abundant information on aerosol type and reveals the aerosol components (e.g., fine- or coarse-mode particles). Hence, measurement and study of lidar ratios under different meteorological conditions can improve the retrieval accuracy of the elastic lidar and quantitatively reveal the primary aerosol optical parameters.

Extensive studies on aerosol lidar ratio have been conducted worldwide in recent years [8,9,10,11]. Landulfo *et al.* [12] obtained a typical lidar ratio value (45 sr) at 532 nm over Sao Paulo, Brazil, through the use of a lidar, a sun photometer, and satellite data during the dry season and suggested that lidar ratios are related to humidity. Bréon [13] conducted passive satellite measurements and found that pollution aerosols have an lidar ratio at 670 nm close to 70 sr, that desert dust values are in the order of 50 sr, and that the lidar ratio at 670 nm of marine aerosols is close to 25 sr; these results indicate the presence of different lidar ratios in different aerosol types. For Saharan dust, Groß *et al.* [14] found that mixtures of biomass-burning aerosols and dust show wavelength independent values of lidar ratios between 57 and 98 sr, respectively. Wiegner *et al.* [15] gave some numerical models for dust-lidar ratio, which make the complex relation between microphysics and lidar ratio clear. Groß *et al.* [16] pointed out that, until now, the mechanism and magnitude of dust aging have been unknown in addition to whether and how they influence the optical properties of dust. Moreover, Ansmann *et al.* [17] reported lidar ratios ranged from 55 ± 5 sr (Munich) to 60 ± 5 sr (Leipzig) in the main Volcanic ash layer, which, measured over Germany, was monitored with multiwavelength Raman lidars and a sun photometer at Leipzig and Munich. Many studies have also been conducted on particle lidar ratio in China. For example, Liu *et al.* (2002) measured the lidar ratio at 532 nm of Asian dusts (42–55 sr) with a high-spectral-resolution lidar and combined Raman-Mie backscatter lidar values [18]. Tesche *et al.* [7] reported that Beijing lidar ratios (clean conditions) accumulate from 30–45 sr with a mean value of 38 ± 7 sr, and the lidar ratios (haze and smog) of the Pearl River Delta (PRD) have larger values between 40 and 55 sr with a mean value of 47 ± 6 sr. These results imply that different pollution levels exert a significant influence on the lidar ratio. A study on the aerosol lidar ratio at 523 nm in Hong Kong was conducted by utilizing a combination of a micro-pulse lidar and a moderate-resolution imaging spectroradiometer [3]. The mean aerosol lidar ratio for the entire period was 29.1 ± 5.8 sr, with a minimum of 18 sr in July 2003 and a maximum of 44 sr in March 2004. The researchers found that the local lidar ratios were considerably affected by the South Asian monsoon and meteorological conditions. The preceding analyses indicate that lidar ratio is severely affected by pollution levels, aerosol types, meteorological factors, and so on. Therefore, the measurement and study of lidar ratio under different pollution levels and atmospheric conditions, and the investigation of the relationship of lidar ratio with other correlation parameters, can improve the retrieval accuracy of regional ground and global satellite lidars. It should also be noted that long-term measurements and studies on lidar ratio in Central China are limited because of the lack of ground-based observations.

To assess lidar ratio in Central China quantitatively, we investigated the variations in nightly particle lidar ratio from July 2013 to May 2015 with a self-developed Raman lidar at Wuhan University in urban Wuhan, Central China. Highly, moderately, and lightly polluted cases were analyzed. The seasonal variation characteristics of lidar ratio and the relationship of lidar ratio with wind were also investigated in detail. Statistical analyses were conducted to provide a comprehensive understanding of aerosol climatology in this region.

## 2. Location, Instrument, and Methodology

Aerosol characteristics are mainly determined by geographical and climatological features as well as their sources of observation sites. A Raman lidar system operates at the top of a building of the State Key Laboratory of Information Engineering in Surveying, Mapping, and Remote Sensing (30°32′ N, 114°21′ E and 30 m above sea level) at Wuhan University, Wuhan, Hubei province, China, which is an urban area. Being the largest city in central China, with the Yangtze River and the Han River traversing across the main city, this region experiences a typical north subtropical humid monsoon climate, with an annual average temperature of 15.8–17.5 °C and an annual average rainfall of 1050–2000 mm [19,20]. Wuhan is a densely populated city with an area of only over 8494 km^2^ and a population of 10.38 million in 2014. It is one of the most rapidly developing and heavily industrialized regions in Central China. The Raman lidar allows for the determination of the profiles of the particle backscatter coefficient, the extinction coefficient, and the lidar ratio at nighttime [21,22].

Raman lidar has been measured since July 2013. The site, which is at the rooftop of a building, is 28 m above ground level. The sampling rate of the Raman lidar is 20 MHz, which corresponds to a vertical spatial resolution of 7.5 m. The Raman lidar pulse repetition rate is 10 Hz with an energy of 200 mJ, and the wavelength is 532 nm (Nd:YAG) with a pulse width of ~10 ns. We described the Raman lidar system in detail in an earlier study [23]. The backscattering signals of Raman lidar are recorded every 1 min. Data from 4 July 2013 to 21 May 2015, except for a maintenance period from May 2014 to November 2014, were used in this study. Our Raman lidar is a self-developing lidar, and many measurements are only for signal tuning. A total of 74 measurements days were generated in this period. However, we only used these stable observations with high signal-to-noise ratio (*i.e.*, the range of N_2_-Raman signal is larger than 8 km) during the measuring days, which is important for choosing an appropriate reference altitude in the retrieval of an accurate particle backscatter coefficient [22]. Thus, only 56 days were applied to retrieve the lidar ratio in this study. The monthly numbers of selected days are shown in Table 1. The Raman lidar signals were averaged within a time window of 20 min for use in the retrieval.

The particle extinction coefficient and backscatter coefficient at 532 nm can be retrieved directly through the N_2_-Raman signal and by combining the Mie and N_2_-Raman signal [22,24,25]. To obtain an accurate particle extinction coefficient, the least square method is used with 50 points (*i.e.*, 375 m) to reduce noise influences. Lidar ratio at 532 nm can be obtained by

(1) $$S_{p}(\lambda_{L},r)=\alpha_{p}\left(\lambda_{L},r\right)/\beta_{p}\left(\lambda_{L},r\right)$$

where $\alpha_{p}\left(\lambda_{L},r\right)$ and $\beta_{p}\left(\lambda_{L},r\right)$ are the particle extinction and backscatter coefficients at wavelength $\lambda_{L}$, respectively; AOD, which form the lidar-derived *αp*, is defined as the integral of particle extinction coefficient along the optical path from *r_1_* to *r_2_* and can be expressed as:

(2) $$AOD(\lambda_{L},r)=\int_{r_{1}}^{r_{2}}\alpha_{p}\left(\lambda_{L},r\right)dr$$

The AOD value in this study was only 0–3 km. The particle extinction coefficient below a height of 0.8 km is calculated from the particle backscatter coefficient multiplied by the lidar ratio determined for the lowest measurement height. The missing range above 3 km, where some of the aerosols reside, leads to an underestimate of the lidar-derived AOD. However, lidar ratio was only determined for heights above 0.8 km because of the effect imposed by incomplete overlap. Moreover, we picked out all the trustworthy lidar ratios that are affected by clouds from 0.8 km to 2 km. To reduce the effects of different smoothing windows between particle extinction and backscatter coefficients, only lidar ratios in homogeneous layers were analyzed in the following study.

## 3. Discussion

### 3.1. Case Analysis

The Raman lidar performed long-term observations at Wuhan University from July 2013 to May 2015. We discuss three classes of pollution each represented by one typical example, namely, heavily, moderately, and lightly polluted cases. The temporal resolution of the retrieved results was 20 min. Five-day backward trajectories for the corresponding cases were demonstrated. The AOD_550_ values at daytime were measured from a co-located sun photometer (CE-318, described in detailed by Wang [19]). The PM_2.5_ (PM_10_), which refers to fine particulate matter that is less than 2.5 (10) micron aerodynamic diameter, were obtained from the closest environmental monitor station. The distance between our lidar site and the station is less than 10 km.

#### 3.1.1. Heavily Polluted Case

**Figure 1:**
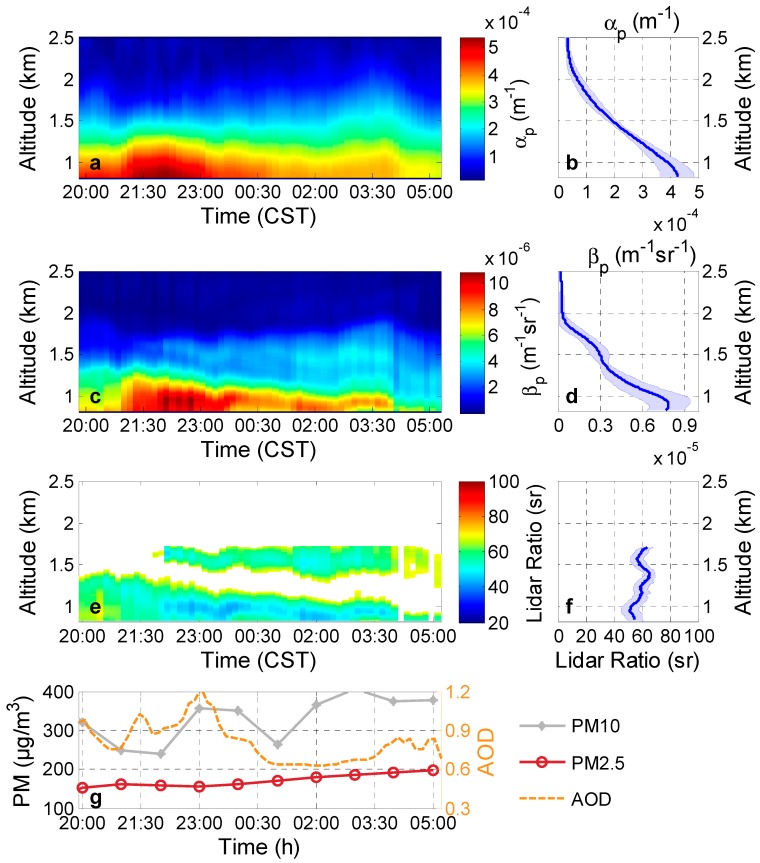
Heavily polluted case: (**a**) particle extinction coefficients (α_p_); (**c**) particle backscatter coefficients (β_p_); respective (**e**) lidar ratios at 532 nm measured in Wuhan on 27–28 October 2013, at 2000–0520 CST; (**b**) mean particle extinction coefficients; (**d**) backscatter coefficients; respective (**f**) lidar ratios from 20:00 to 05:20. The blue shadows on each figures indicate the standard deviations of corresponding values; (**g**) The PM_10_ and PM_2.5_ as well as the AOD retrieved from the Raman lidar.

We selected a typical heavily polluted case under PM_2.5_ (PM_10_) that was larger than 150 (280) μg/m^3^ and approaching 200 (400) μg/m^3^, as shown in Figure 1g. The region was in haze and smog conditions. This case was observed from 20:00 (Chinese Standard Time, CST) on 28 October 2013 to 05:00 the next day. The AOD derived from Raman lidar below 3 km was approximately 0.81 ± 0.15 during nighttime, which can be seen in Figure 1g. The daytime AOD_550_ average value of the sun photometer at 28 October was 1.18 ± 0.11.

The particle extinction coefficient, the particle backscatter coefficient, and the lidar ratio profiles were measured after sunset on 28–29 October. They are shown in Figure 1a,c,e, respectively. The mean profiles of the particle extinction coefficient, the backscatter coefficient, and the lidar ratio during the entire nighttime are respectively plotted in Figure 1b,d,f, and the blue shadows on each figures indicate the standard deviations of corresponding values. Figure 1a,b, show that the particle extinction coefficients under heavily polluted situations are 5.4e-4 m^−1^ in the haze layer from 0.8 km to 1.8 km; this value is slightly smaller than the highest values (close to 8.3e-4 m^−1^) of the haze layer over PRD in South China on 12 October 2004 [26]. The haze layer below 1.8 km has a large particle extinction coefficient (from 5.4e-4 m^−1^ to 1.6e-4 m^−1^) and backscatter coefficient (between 1.1e-05 m^−1^sr^−1^ to 1.7e-06 m^−1^sr^−1^). The mean lidar ratio of the low haze layer (0.8~1.8 km) is about 57 ± 7 sr (43–70 sr), which are slightly higher than the ones observed at PRD (35–59 sr and on average, 46.7 sr) [26]. One-year Raman lidar measurements of particle extinction coefficients and backscatter coefficients at 532 nm were performed from April 2009 to March 2010 at the Global Atmospheric Watch (GAW) station of Shangdianzi in the North China Plain 100 km northeast of Beijing, which showed the lidar ratios with a narrow distribution peaking at 60 sr in the haze layer caused by anthropogenic fine-mode aerosol [27]. The large Ångström exponents (calculated from AODs at 440 and 870 nm) acquired from the sun photometer is about 1.44 (from 1.41 to 1.48) at October 28 during the daytime, indicating that small particles have absolute predominance over Wuhan in this case.

Franke *et al.* point out that the lidar ratio is lowest (20–30 sr) for large maritime particles (low light absorption) and largest (70–100 sr) for small particles (highly absorbing urban haze) [28]. The reason for the high lidar ratio over Wuhan in this case is highly absorbing urban haze accumulated due to a one-month absence of rain. Furthermore, low wind speed (<5 km/h) cannot blow away the thick haze layer. The aerosol in this area results from continuous city construction, high automobile exhaust, low plant coverage, and vast light-absorbing soot generated from domestic cooking, heating, and industrial coal combustion [19].

#### 3.1.2. Moderately Polluted Cases

In this case, Raman lidar measurements were executed on 1–2 December 2013. During the measurement period, westerly to northwesterly air transport prevailed. Co-located sun photometer measurements showed that the 550 nm AODs were approximately 0.61 ± 0.11 (1 December) and 0.47 ± 0.01 (2 December) at daytime. The PM_2.5_ (PM_10_) is about 100 (180) μg/m^3^, which is much less than the PM_2.5_ (PM_10_) in the heavily polluted case, as shown in Figure 1g.

**Figure 2:**
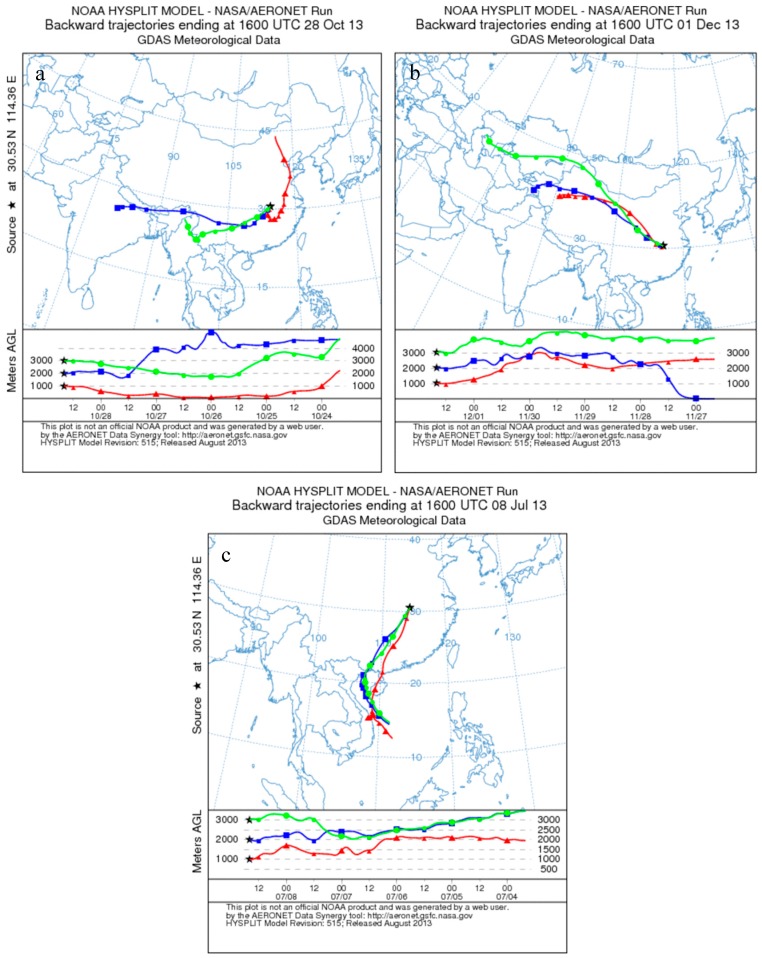
Five-day backward trajectories calculated by HYSPLIT ending at Wuhan at 1000 m (triangles), 2000 m (squares), and 3000 m height (circles) on (**a**) 29 October 2013; (**b**) 2 December; and (**c**) 9 July 2013, at 0000 BST (*i.e.*, UTC + 8) for the corresponding case.

**Figure 3:**
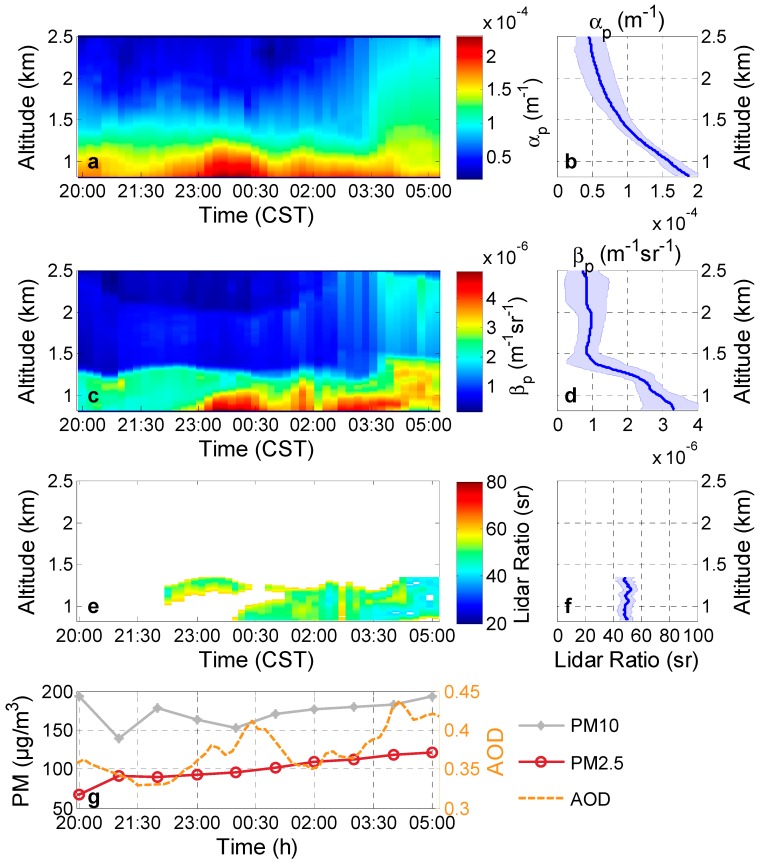
Moderately polluted cases: (**a**) particle extinction coefficients (α_p_); (**c**) particle backscatter coefficients (β_p_); respective (**e**) lidar ratios at 532 nm measured in Wuhan on 1–2 December 2013, at 2000–0520 CST; (**b**) mean particle extinction coefficients; (**d**) backscatter coefficients; respective (**f**) lidar ratios from 20:00 to 05:20. The blue shadows on each figures indicate the standard deviations of corresponding values; (**g**) The PM_10_ and PM_2.5_ as well as the AOD retrieved from the Raman lidar.

Tesche *et al.* [7] observed particle extinction coefficients under the prevailing westerly to northwesterly air flows range from 1e-4 to 4e-4 m^−1^ in most cases and exceed 5e-4 m^−1^ in moderately polluted situations. Figure 2b demostrates five-day backward trajectorie of the moderate pollution case and Figure 3 shows the case measured at Wuhan. The AOD at 532 nm derived from Raman lidar below 3 km was approximately 0.38 ± 0.03 during nighttime, and particle extinction coefficient (backscatter coefficient) was approximately 2.3e-4–1.0e-4 m^−1^ (5.0e-6–1.6e-6 m^−1^sr^−1^) at about 0.8–1.3 km (homogeneous aerosol layer).

According to the backward trajectories in Figure 2b, polluted air originated from the low-industrialized northwest areas of China. Sectional particles might be dust aerosol originating from the Gobi desert. The lidar ratio of long-range transported Saharan dust is equal to 59 sr for 355 nm and 532 nm, with an uncertainty range between ±4 sr and ±10 sr, which was measured by two Raman depolarization-lidar systems at central Europe at the end of May 2008 [29]. The result is similar to the measurement of 53–55 sr, obtained at 355, 532 and 1064 nm from three ground-based Raman lidars and an airborne high-spectral-resolution lidar during SAMUM 2006 in Southern Morocco [30]. However, a low dust lidar ratio (35 ± 5 sr) of the Gobi desert was reported in [21]. In this case, the lidar ratios below 1.3 km are larger than the low lidar ratio of Gobi due to the dust was difficult to become the major influence factors with the low wind speed (less than 5 km/h) near the surface. The lidar ratios were mostly 40–60 sr, with a mean value of 50 ± 5 sr at 0.8 km to 1.3 km in this case. In the moderately polluted case, the particle component might include anthropogenic particles and dust particles. The relatively large Ångström exponents (1.22) measured by the sun photometer at daytime indicate the important influence of fine mode particles on the particle optical properties.

#### 3.1.3. Lightly Polluted Case

**Figure 4:**
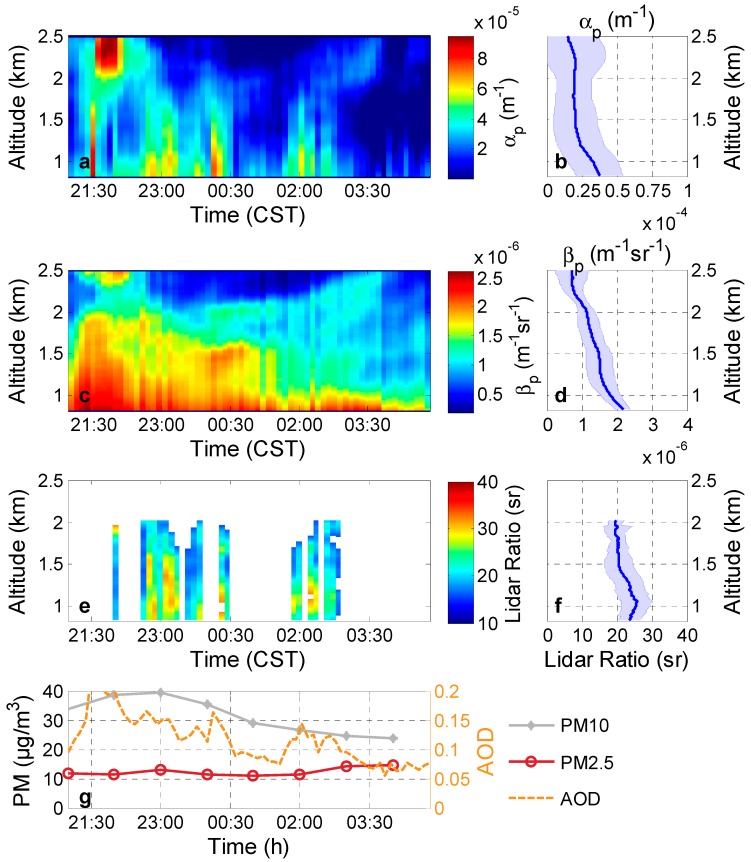
The lightly polluted case: (**a**) particle extinction coefficients (α_p_); (**c**) particle backscatter coefficients (β_p_); respective (**e**) lidar ratios at 532 nm measured in Wuhan on 7–8 July 2013, at 2000–0520 CST; (**b**) mean particle extinction coefficients; (**d**) backscatter coefficients; respective (**f**) lidar ratios from 20:00 to 05:20. The blue shadows on each figures indicate the standard deviations of corresponding values; (**g**) The PM_10_ and PM_2.5_ as well as the AOD retrieved from the Raman lidar.

Almost clear conditions were observed on 8 July 2013 (Figure 2c and Figure 4). PM_2.5_ (PM_10_) less than 10(40) μg/m^3^ is shown in Figure 4g. A heavy rain continued for three days before the case (*i.e.*, 5 July, 6 July, and 7 July) and the amount of precipitations were 35.05 mm, 24.89 mm, and 77.98 mm for each day. The anthropogenic influence was also considerably low according to the backward trajectories in Figure 2c with high wind speed (mean value is 14 km/h and max value is 32 km/h).

The AOD at 532 nm below 3 km retrieved by the Raman lidar was about 0.12 ± 0.04, and the mean particle extinction coefficient was about 2.4e-5 m^−1^ above 0.8 km. The backward trajectory crossed South China Sea and was obviously affected by the clean south wind of the summer monsoon. The lidar ratios of this case were extremely low, with values of 16–32 sr (22 ± 4 sr) at 0.8–2 km.

Maritime aerosol (lidar ratio less than 20 sr) [31] could be a possible source contributing to the low lidar ratios, as demonstrated in Figure 2c. This case suggested that excellent weather frequently appeared in the summer caused by the clean South Asian summer monsoon. We have to note that some particle extinction coefficients cannot be retrieved because the aerosols at some altitude are extremely low. Thus, the lidar ratios at these regions with extremely low aerosol in this case were removed.

### 3.2. Long-Term Analysis

**Figure 5:**
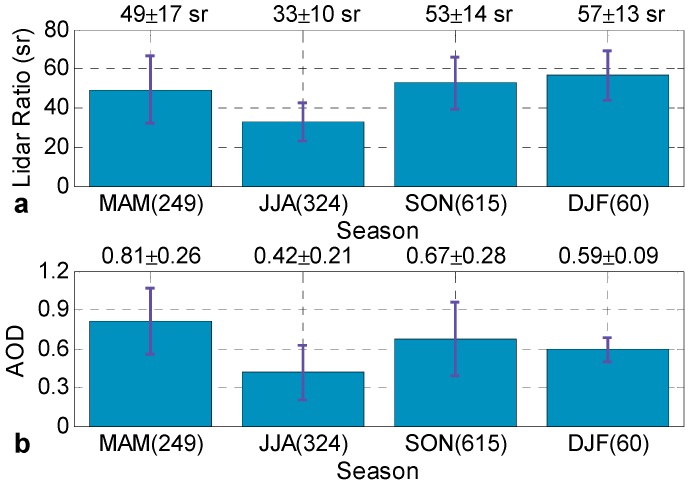
Seasonal (**a**) mean aerosol lidar ratios and (**b**) AODs at 532 nm derived from Raman lidar measurements in Wuhan between July 2013 and May 2015. The numbers in each bracket represent the number of available results of the corresponding season. The error bars represent the corresponding standard deviations.

Figure 5 provides an overview of the measured lidar ratios and AODs in Central China. Statistical analysis of data derived from Raman Lidar at no-rain nighttime (*i.e.*, 20:00–05:00 BST) from July 2013 to May 2015. The temporal resolution of the retrieved results was 20 min, and approximately 1239 results with 20-min-average were available at all times. Spring, summer, fall, and winter dated from March to May (MAM), June to August (JJA), September to November (SON), and December to February (DJF) of the following year, respectively. The numbers of available results were 240, 324, 615, and 60 in MAM, JJA, SON, and DJF, respectively. It should be noted that the statistical results may not represent completely the true quarterly value because of the inadequate lidar measuring data.

Figure 5a shows the seasonal means of lidar ratio at 532 nm during the entire study period. The seasonal mean lidar ratios were 49 ± 17 sr, 33 ± 10 sr, 53 ± 14 sr, and 57 ± 13 sr for MAM, JJA, SON, and DJF, respectively. These lidar ratio values are slightly higher than those measured by combination of micro-pulse LIDAR and MODIS in Hong Kong, a coastal city in South China, which exhibited 34.4 ± 6.9 sr, 27 ± 5.8 sr, 28.3 ± 4.8 sr, and 31.1 ± 6.0 sr, respectively, between 1 May 2003 and 30 June 2004 [3]. The abundant maritime aerosol might contribute to the lower value for Hong Kong. Several similar conclusions have been obtained before. Several higher lidar ratios were measured by a compact Raman lidar in Beijing, China, from 15 to 31 December 2007, and the mean lidar ratio was 60.8 ± 13.5 sr in a moderate-pollution episode [32]. The quarterly variation in lidar ratios is related to anthropogenic pollution and monsoon climate. The large lidar ratios in spring, autumn and winter are associated with the contribution of local industrial pollutants and straw burning transported by the prevailing northerly winds. On the contrary, the low lidar ratio values in summer are characteristic of the dominance of mixed oceanic and local anthropogenic aerosols. Moreover, the abundant rainfall in summer also contributes to the low lidar ratio values. Thus, these results demonstrate the essential dependence of lidar ratio on the Asiatic monsoon, which is the dominant circulation feature over East Asia.

Figure 5b shows the seasonal means of AODs at 532 nm below 3 km. The seasonal mean AODs are 0.80 ± 0.25, 0.42 ± 0.21, 0.66 ± 0.27, and 0.59 ± 0.09 for MAM, JJA, SON, and DJF, respectively. These AOD values are lower than the seasonal mean AODs at 500 nm measured by a sun photometer at Wuhan, which were 1.03 ± 0.52, 1.13 ± 0.71, 1.07 ± 0.75, and 1.05 ± 0.60 from 2007–2013, respectively [19]. One of the reasons for the lower values is that the sun photometer measured at daytime, whereas the Raman lidar operated at nighttime when anthropogenic aerosol was lower because the location of the Raman lidar was in the urban centers and near arterial traffic in Wuhan. Furthermore, the Raman lidar results were only from 0–3 km, whereas the sun photometer results were from throughout the atmosphere. Another small effect can be expected from the different wavelengths. Hazes frequently occur in spring and autumn [19], which results in a higher AOD. This seasonal variation in AOD is due to the contributions of pollutant particles emitted from local sources and particles brought by long-range transport.

**Figure 6:**
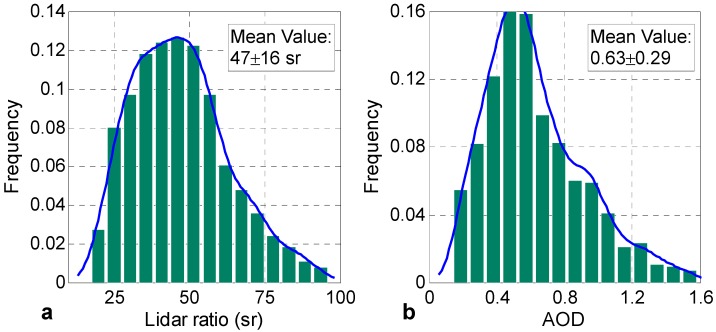
Frequency distribution of (**a**) lidar ratios and (**b**) AODs; the blue solid lines represent the fitting curves.

Figure 6a,b present the frequency distribution of lidar ratios and AODs (0–3.0 km) from July 2013 to May 2015. They show that most of the lidar ratios (AODs) are less than 60 sr (0.9), with a mean value of 47 ± 16 sr (0.62 ± 0.28). Some large lidar ratios and AODs indicate that the urban haze type with a strong extinction capability plays an important role in Wuhan.

**Figure 7:**
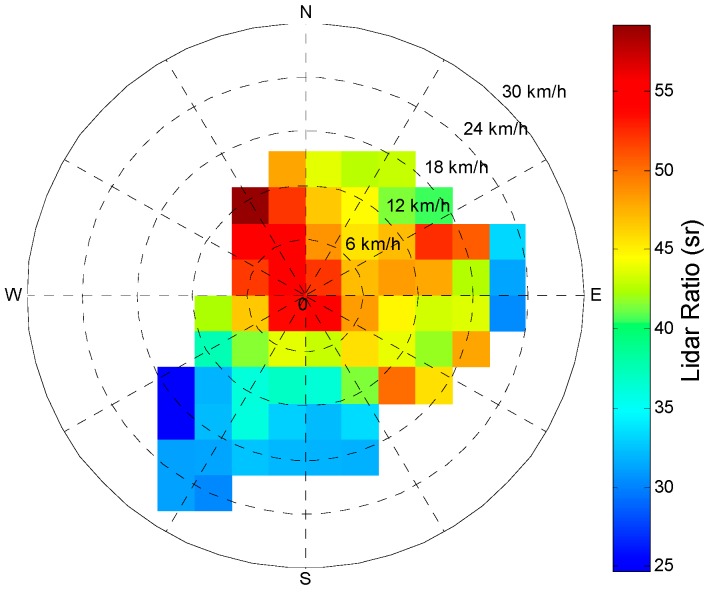
Variation in aerosol lidar ratios as a function of wind direction (°) and speed (km/h).

Assessing the relationships between lidar ratio and numerous potential pollutant sources around the city is important to comprehensively investigate the characteristics of lidar ratios in Wuhan. Figure 7 displays the lidar ratio dependence on wind direction and speed. The center of the circle indicates that lidar ratio (52 ± 15 sr) is minimally influenced by low wind speed (less than 6 km/h) regardless of the wind direction in the measured location; these values represent the normal values of the city. This finding may be related to the dominance of local urban or industrial emissions with strong absorption near the Raman lidar site. The lidar ratio values in Wuhan are lower (34 ± 1 sr) under prevailing southwesterly and southerly wind (wind direction range: 120° to 240°) when wind speed is higher than 12 km/h. The extremely low values (~25 sr) also appear under these conditions. These relatively low values are primarily caused by the combination of local pollution aerosol with clean aerosols from the southwesterly mountain, which is an undeveloped region with a large forest. The other reason is similar to that for the lightly polluted case in Figure 4 (caused by the Asian summer monsoon). Several large lidar ratio values (up to 55 sr) are associated with strong northern winds. The pollution in the north region of China is the worst, and haze frequently occurs with high PM_2.5_, especially in autumn and winter. The easterly low value (about 35 sr) may be affected by the maritime aerosol transport from the East China Sea.

## 4. Conclusions

The long-term lidar ratio at 532 nm was measured and studied with a Raman lidar from July 2013 to May 2015 at Wuhan University. We analyzed highly, moderately, and lightly polluted cases. The five-day backward trajectories for the corresponding cases were demonstrated. The profiles of the particle extinction coefficients and backscatter coefficients and the respective lidar ratios were presented. We found that the lidar ratios exhibit large differences under different atmospheric conditions. The mean values are approximately 57 ± 7 sr, 50 ± 5 sr, and 22 ± 4 sr under the three cases with different pollution levels. The haze layer below 1.8 km has a large particle extinction coefficient (from 5.4e-4 m^−1^ to 1.6e-4 m^−1^) and backscatter coefficient (between 1.1e-05 m^−1^sr^−1^ and 1.7e-06 m^−1^sr^−1^) in the highly polluted case, which indicates a prominent effect of vast fine particles on optical properties. The extremely small lidar ratio (16–32 sr) in the clear case indicates that the summer South Asia monsoon from the South China Sea contributes clear aerosol with coarse aerosol particles; this lidar ratio value is much smaller than the lidar ratios purely caused by anthropogenic haze. The quarterly variation in lidar ratios is related to anthropogenic pollution and monsoon climate. The large lidar ratios in spring (49 ± 17 sr), autumn (53 ± 14 sr), and winter (57 ± 13 sr) are associated with the contribution of local industrial pollutants and straw burning transported by the prevailing northerly winds. The low lidar ratio value (33 ± 10 sr) in summer is a characteristic of the dominance of mixed oceanic and local anthropogenic aerosols. Thus, this study demonstrated the essential dependence of the lidar ratio on the Asiatic monsoon, which is the dominant circulation feature over East Asia. The correlations between lidar ratio and wind also imply that wind has some important influences on lidar ratios. In summary, large values of lidar ratio correspond well with weak winds and strong northerly winds, whereas significantly low lidar ratio values are associated with prevailing southwesterly and southerly wind.

Our analysis indicated that lidar ratio is a complicated parameter that is difficult to quantitatively characterize. Future long-term measurements for lidar ratio values using Raman lidar and other sensors are still necessary. The relationship between lidar ratio and other optical parameters or synoptic conditions requires in-depth study. The obtained aerosol lidar ratios also need to be compared with those from other populous cities in the world for widespread knowledge on the macroscopic and microscopic characteristics of aerosols.

## Figures and Tables

**Table 1:** Monthly day numbers of Raman lidar measurements in Wuhan from 4 July 2013 to 21 May 2015.

Month	Measuring Day Numbers
July 2013	11
August 2013	6
September 2013	1
October 2013	11
November 2013	6
December 2013	1
March 2014	4
April 2014	2
December 2014	2
April 2015	9
May 2015	3
